# Clinical insights into mixed *Candida* and bacterial bloodstream infections: a retrospective cohort study

**DOI:** 10.1128/spectrum.01684-25

**Published:** 2025-09-04

**Authors:** Ho-Yin Huang, Ying-Chi Lin, Po-Liang Lu, Ya-Ling Wang, Tun-Chieh Chen, Ko Chang, Shang-Yi Lin

**Affiliations:** 1 Department of Pharmacy, Kaohsiung Medical University Hospital, Kaohsiung Medical University 38023, Kaohsiung, Taiwan; 2 School of Pharmacy, College of Pharmacy, Kaohsiung Medical University 38023, Kaohsiung, Taiwan; 3 Master/Doctoral Degree Program in Toxicology, College of Pharmacy, Kaohsiung Medical University 38023, Kaohsiung, Taiwan; 4 Division of Infectious Diseases, Department of Internal Medicine, Kaohsiung Medical University Hospital 89234, Kaohsiung, Taiwan; 5 College of Medicine, Kaohsiung Medical University 38023, Kaohsiung, Taiwan; 6 Department of Internal Medicine, Kaohsiung Municipal Hsiao-Kang Hospital, Kaohsiung Medical University 38023, Kaohsiung, Taiwan; 7 Graduate Institute of Clinical Medicine, Kaohsiung Medical University 38023, Kaohsiung, Taiwan; 8 Department of Laboratory Medicine, Kaohsiung Medical University Hospital 89234, Kaohsiung, Taiwan; University of Maryland School of Medicine, Baltimore, Maryland, USA

**Keywords:** candidemia, bloodstream infections, co-infection, mixed *Candida*/bacterial BSIs, mortality, EQUAL score

## Abstract

Mixed bloodstream infections (BSIs) involving *Candida* and other bacterial pathogens present significant diagnostic and therapeutic challenges. The incidence of these co-infections has been increasing. However, the limited literature on this topic necessitates a deeper understanding of its epidemiological and clinical characteristics. This retrospective study evaluated adult patients with candidemia across three hospitals in Taiwan between January 2014 and December 2020. Mixed *Candida*/bacterial BSIs were defined as the concurrent isolation of bacterial species from any set of blood cultures within 1 week of the initial *Candida* culture. The clinical features and 30-day mortality of mixed *Candida*/bacterial BSIs were investigated. Among 766 patients with candidemia, 193 (25.2%) had mixed *Candida*/bacterial BSIs. The 30-day mortality for mixed infections was significantly higher than for monomicrobial candidemia (62.7% vs. 51.7%, *P* = 0.009). The mortality rates and intervals between *Candida* and bacterial blood culture isolation had a negative correlation (*P* = 0.004). In the mixed *Candida*/bacterial BSI cases, the most common bacteria included coagulase-negative staphylococci, *Enterococcus* spp., and *Klebsiella* spp. Co-infection with vancomycin-resistant *Enterococcus* had a significantly higher mortality rate than mono-candidemia (*P* = 0.015). Significant predictors of 30-day mortality included high Pitt score, high sequential organ failure assessment (SOFA) score, inappropriate empiric antibiotics, and lack of adherence to the European Confederation of Medical Mycology Quality of Clinical Candidaemia Management (EQUAL) score. In conclusion, mixed *Candida*/bacterial BSIs are associated with high mortality and severe clinical conditions. Empirical antibiotic strategies and tailored antifungal treatments, aligned with clinical guidelines, have significantly improved patient outcomes.

**IMPORTANCE:**

Mixed bloodstream infections with *Candida* and bacteria are serious and deadly. This study demonstrated that *Candida*/bacterial bloodstream infections (BSIs) accounted for 25.2% of all candidemia cases and were associated with significantly higher 30-day mortality compared with monomicrobial candidemia (62.7% vs 51.7%). Patients with mixed BSIs exhibited more severe clinical conditions, a higher rate of inappropriate antibiotic use, and a greater prevalence of multidrug-resistant organisms. The study further demonstrated that adherence to antifungal treatment recommendations (EQUAL *Candida* score) and timely initiation of appropriate empirical antibiotics were associated with improved survival. These findings fill a critical gap in the literature and provide important insights into the management of mixed *Candida*/bacterial BSIs, with implications for optimizing diagnostic and therapeutic strategies in clinical practice.

## INTRODUCTION

Bloodstream infections (BSIs) are a major cause of morbidity and mortality among hospitalized patients, with *Candida* species emerging as major pathogens in healthcare-associated BSIs (1). Recently, the incidence of candidemia has been increasing and is associated with mortality rates as high as 60%, extended hospital stays, and increased medical costs, presenting a substantial public health challenge (2–4).

The complexity of managing candidemia is further exacerbated by mixed *Candida*/bacterial infections, accounting for >20% of all candidemia episodes and exhibiting a growing trend (5–9). The risk factors for these mixed infections include compromised immune systems, intensive care unit stay, prolonged hospitalization, and use of broad-spectrum antibiotics (6, 9, 10). The coexistence of bacterial pathogens and *Candida* significantly challenges clinical management and adversely affects patient outcomes (5–9). However, research on mixed *Candida*/bacterial BSIs are limited, primarily focusing on comparisons with monomicrobial candidemia (mono-candidemia) regarding risk predictors, treatment methods, and clinical outcomes. These studies are often limited by their focus on specific patient populations and small sample sizes (5–9). The epidemiological characteristics and outcome indicators specific to mixed infections have seldom been comprehensively addressed. Therefore, this study aimed to determine the frequency and the 30-day mortality predictors in adult patients with mixed *Candida*/bacterial BSIs.

## MATERIALS AND METHODS

### Study design and hospital setting

This multicenter, retrospective, observational cohort study was conducted at three institutions affiliated with Kaohsiung Medical University in Southern Taiwan, including a tertiary medical center and two regional hospitals, with a combined total of 2,400 beds. This study was reviewed and approved by the Institutional Review Board of Kaohsiung Medical University Hospital (IRB no. KMUHIRB-E(I)−20240161).

### Patient population and definition

Adult patients (aged ≥18 years) who had at least one positive blood culture report for a *Candida* species with clinical symptoms or signs of infection between January 2014 and December 2020 were included. Only the first episode was considered in patients with multiple candidemia episodes. Mixed *Candida*/bacterial BSIs were defined as the concurrent isolation of bacterial species from the same or different blood culture sets within 1 week before or after the candidemic episode (5). The microorganisms considered probable contaminants included *Bacillus* spp., *Corynebacterium* spp. (excluding *Corynebacterium jeikeium*), *Lactobacillus* spp., and *Propionibacterium* spp. If isolated from two distinct venipuncture samples and associated with a central venous catheter (CVC), coagulase-negative staphylococci (CoNS), or viridans group streptococci were considered the probable pathogens (11). A review process, including clinical symptoms of infection, was conducted to exclude contamination, with the involvement of two independent clinicians or infectious diseases specialists, and the discrepancies were resolved by consensus.

Demographics, clinical and microbiological characteristics, and use of antibiotics and antifungal agents were gathered from the medical records of eligible patients. The Charlson comorbidity index (CCI) was used to evaluate comorbidities (12). Neutropenia and thrombocytopenia were defined as absolute neutrophil and platelet counts of <500 cells/mm^3^ and <150,000/mm^3^, respectively. Steroid therapy involved the administration of corticosteroids > 20 mg/day for 2 weeks. Septic shock was defined as a systolic blood pressure <90 mmHg or the need for vasopressors. Disease severity at the onset of candidemia was evaluated using the sequential organ failure assessment (SOFA) and Pitt bacteremia scores (13, 14). Previous use of antibiotics or azoles was recorded within 30 days before the initial blood culture. The sources of candidemia followed the US Centers for Disease Control and Prevention definitions, and source control was defined as removing a focus excluding catheter extraction (15). The appropriateness of empirical antibiotic therapy depends on the *in vitro* susceptibility of the pathogens to the administered agents. Multidrug-resistant organisms (MDROs) are defined as microorganisms, predominantly bacteria, that are resistant to one or more classes of antimicrobial agents (16). The European Confederation of Medical Mycology Quality of Clinical Candidaemia Management (EQUAL) score was used to measure guideline adherence for diagnosing and managing candidemia (17). The outcome measured was the 30-day mortality.

### Microbiological studies

Our institution used the BacT/ALERT 3D (BioMérieux, Durham, NC, USA) instrumented automated blood culture system. All isolates were identified to the species level. Before July 2015, positive blood cultures were first examined microscopically and then confirmed using the ID 32 C system (bioMérieux, Durham, NC, USA). After July 2015, the Bruker Biotyper MALDI-TOF MS system (version 3.0; Bruker Daltonik GmbH, Leipzig, Germany) was used for routine identification. According to the manufacturer’s instruction, the identification cut-off score was interpreted as follows. A score of ≥2.000 indicated species-level identification, between 1.700 and 1.999 genus-level identification, and <1.700 no identification (18). Antimicrobial susceptibility tests were performed using the Vitek 2 system (bioMérieux, Marcy l'Etoile, France; software version 5.04 in 2014, upgraded to 7.01 in June 2016, and to 8.01 in October 2018) and Sensititre Yeast One panel (Trek Diagnostic Systems, Cleveland, OH, USA). The results were interpreted according to the Clinical and Laboratory Standards Institute (CLSI) Performance Standards for Antimicrobial Susceptibility Testing (19).

### Statistical analysis

Statistical analyses were performed using SPSS version 20 (IBM Corp., Armonk, NY, USA). Continuous variables with normal distribution are shown as mean and standard deviation and analyzed using the Student’s *t*-test. Categorical variables are displayed as numbers and analyzed using the χ or Fisher’s exact test, as needed. The Kaplan–Meier method was used to estimate event-time distributions, and the Mantel–Haenszel test was used to assess trends in blood culture data and mortality. Variables that were significant in the univariable analysis were included in a multivariable logistic regression analysis using backward stepwise algorithm to identify the mortality-associated factors. All tests were two-sided, with a significance level of *P* < 0.05.

## RESULTS

Across 7 years, 813 adult patients with clinically significant candidemia were identified. Of these, 36 were excluded because of incomplete data; hence, 777 eligible participants were identified. After excluding 11 cases with contaminated microorganisms, 193 patients (25.2%) had mixed *Candida*/bacterial BSIs, and the remaining 573 patients were diagnosed with mono-candidemia (Fig. S1).

**Fig 1:**
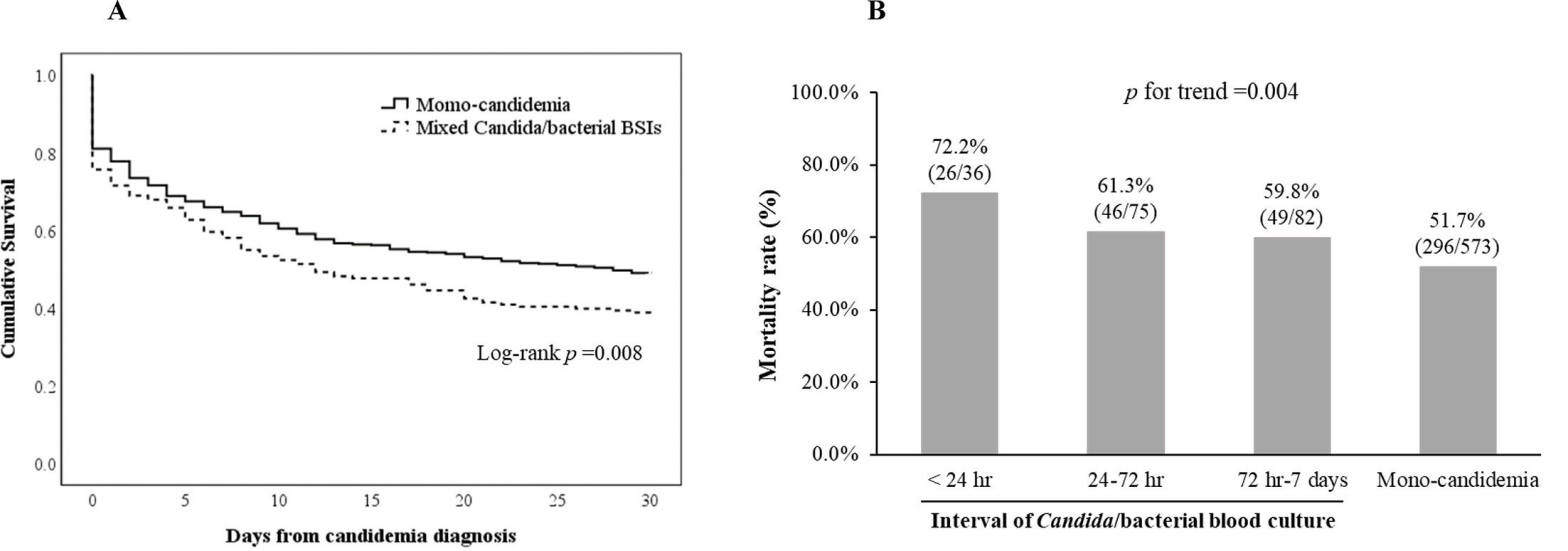
(**A**) Kaplan–Meier estimates of survival in patients with mixed *Candida*/bacterial BSIs and mono-candidemia. (**B**) Comparison of the mortality rates in different intervals of *Candida* and bacterial blood culture isolation.

The clinical characteristics of 766 patients with candidemia are shown in Table S1. Patients with mixed *Candida*/bacterial BSIs exhibited higher incidences of septic shock and thrombocytopenia, along with elevated SOFA and Pitt bacteremia scores. The overall 30-day mortality rate of patients with candidemia was 54.4% (417/766). The mortality rate was significantly higher in patients with mixed *Candida*/bacterial BSIs than in those with mono-candidemia (62.7% vs 51.7%; *P* = 0.009). The Kaplan–Meier method indicated superior survival in the mono-candidemia group compared with that in the mixed *Candida*/bacterial BSIs group (*P* = 0.008; Fig. 1A). A negative relationship was identified between mortality rates and time interval between *Candida* infection and bacterial blood culture isolation (*P* = 0.004; Fig. 1B).

**Fig 2:**
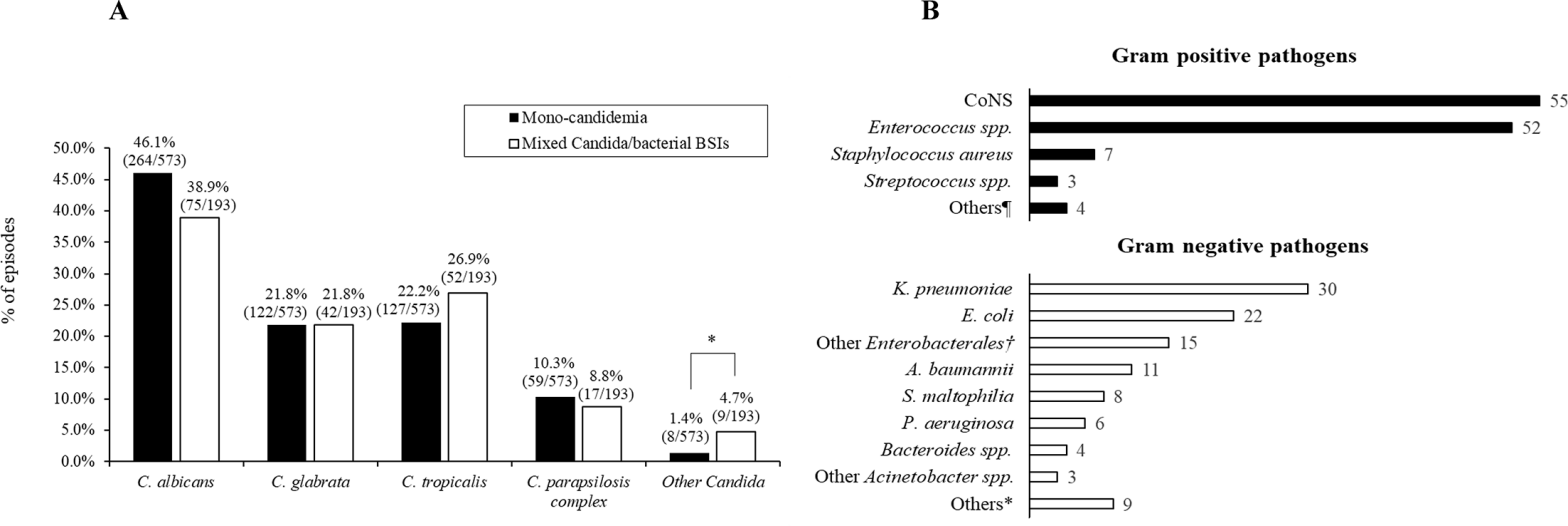
Distribution of the (**A**) *Candida* species isolated from patients with candidemia and (**B**) bacterial species and numbers isolated from mixed *Candida*/bacterial BSIs. Other *Candida* species included *C. dubliniensis* (2), *C. guilliermondii* (2), *C. lusitaniae* (4), *C. haemulonii* (3), *C. intermedia* (1), *C. pelliculosa* (2), *C. ciferrii* (1), *C. nivariensis* (1), *C. famata* (1). * means *p* < 0.05, ¶ *Arthrobacter* species (1), *Corynebacterium jeikeium* (1), *Dermacoccus nishinomiyaensis*/*Kytococcus sedentarius* (1), and Viridans group streptococci (1). † *Citrobacter freundii* (1), *Enterobacter* spp. (8), *Proteus mirabilis* (2), and *Serratia marcescens* (4). * *Achromobacter xylosoxidans* (1), *Brevundimonas diminuta*/*vesicularis* (1), *Burkholderia cepacia* (1), *Elizabethkingia meningoseptica* (1), *Ralstonia pickettii* (1), *Sphingomonas paucimobilis* (1), and *Veillonella parvula* (2). CoNS, coagulase-negative staphylococci. The black bar indicates the number of Gram-positive bacteria, and the white bar indicates the number of Gram-negative bacteria.

**Fig 3:**
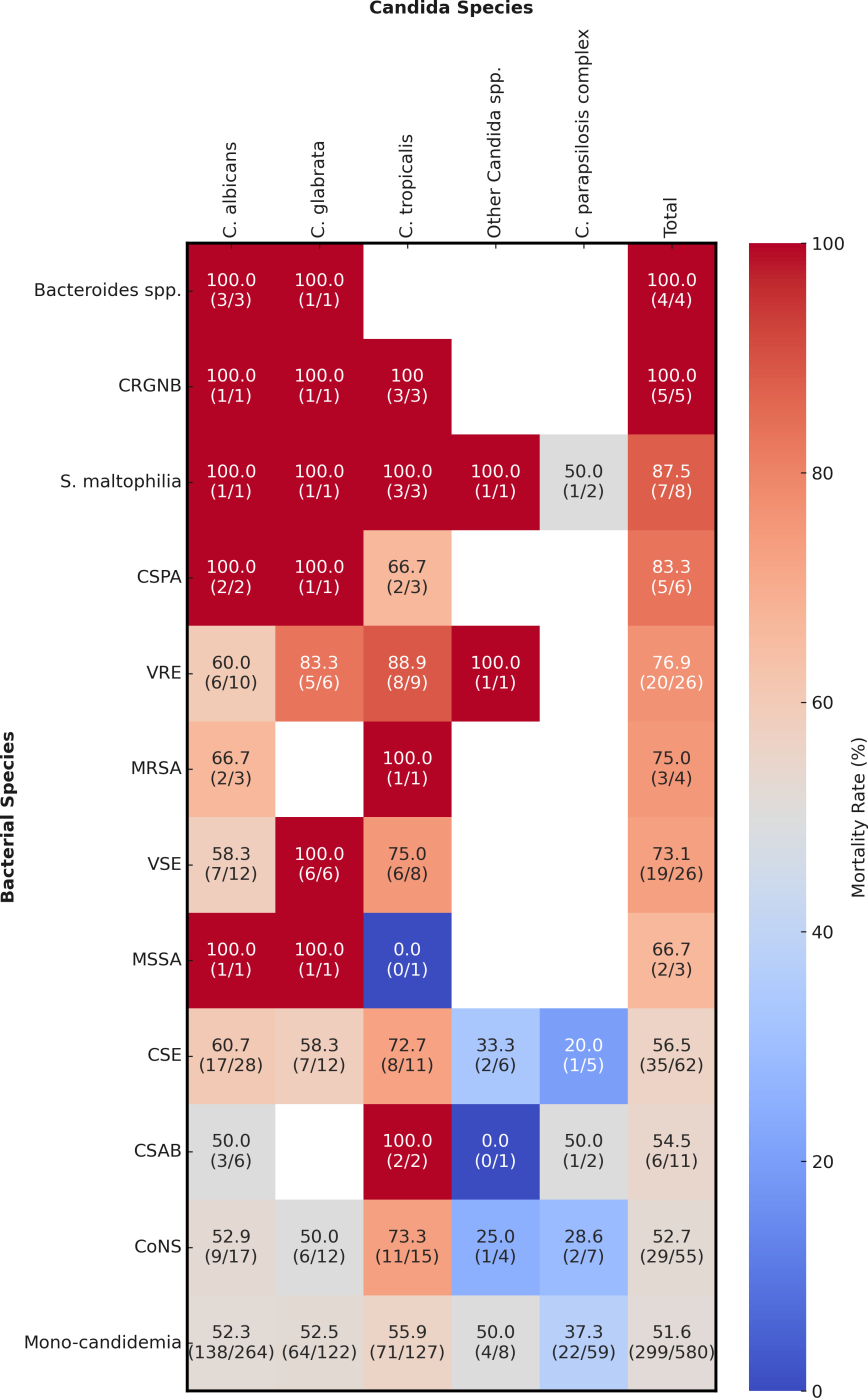
Mortality rates in different combinations of *Candida* and bacterial species. The numbers in the heatmap show the mortality rate as a percentage. The parentheses contain the number of deaths over the total infected patients for each combination of bacterial species (rows) and *Candida* species (columns). CRGNB, carbapenem-resistant gram-negative bacteria; CSPA, carbapenem-susceptible *Pseudomonas aeruginosa*; VRE, vancomycin-resistant *Enterococcus*; MRSA, methicillin-resistant *Staphylococcus aureus*; VSE, vancomycin-susceptible *Enterococcus*; MSSA, methicillin-susceptible *Staphylococcus aureus*; CSE, carbapenem-susceptible *Enterobacterales*; CSAB, carbapenem-susceptible *Acinetobacter baumannii*; CoNS, coagulase-negative staphylococci

Figure 2 shows the distribution of *Candida* species from patients with candidemia and bacterial species from those with mixed *Candida*/bacterial BSIs. The frequency of major *Candida* species, including *C. albicans, C. glabrata, C. tropicalis*, and *C. parapsilosis* complex, was consistent across both groups. However, the prevalence of less common *Candida* species, such as *C. guilliermondii* and *C. lusitaniae*, differed significantly between the two groups (*P* = 0.018; Fig. 2A). Among the 229 bacterial isolates, 121 (52.8%) were Gram-positive bacteria. The most frequently identified species were CoNS (24.0%), *Enterococcus* spp. (22.7%), and *Klebsiella* spp. (13.1%). Additionally, the data set included 35 MDROs: four methicillin-resistant *Staphylococcus aureus* (MRSA), 26 vancomycin-resistant *Enterococcus* (VRE), and five multidrug-resistant gram-negative bacteria (MDR-GNB), including two carbapenem-resistant *Acinetobacter baumannii*, two carbapenem-resistant *Klebsiella pneumoniae*, and one carbapenem-resistant *Pseudomonas aeruginosa*. Candidemia cases associated with MDROs had increased mortality rates (*P* = 0.021), with the highest mortality rate being 91.7% for *C. tropicalis* (Fig. S2). A heatmap depicting the mortality rates across various combinations of *Candida* and bacterial species is shown in Fig. 3. VRE was associated with significantly higher mortality rate compared with cases of mono-candidemia (*P* = 0.015).

The clinical features of 193 patients with mixed *Candida*/bacterial BSIs are shown in Table 1. In multivariable logistic regression analysis, high Pitt score and high SOFA score were risk factors for 30-day mortality, whereas the empirical use of appropriate antibiotics and high EQUAL score were protective independent predictors for 30-day mortality (Table 2).

**TABLE 1:** Clinical characteristics of 193 patients with mixed *Candida*/bacterial bloodstream infections^*f*^

	Total *N* = 193, (%)		30-day mortality				
Characteristic			Survivors *N* = 72, (%)		Non-survivors *N* = 121, (%)		*P*-value
Demographics							
Age (year), mean ± SD	66.3 ± 15.34		65.8 ± 15.8		66.5 ± 15.2		0.748
Male, sex	99	(51.3)	35	(48.6)	64	(52.9)	0.655
Surgical ward	27	(14.0)	14	(19.4)	13	(10.7)	0.132
Medical ward	39	(20.2)	19	(26.4)	20	(16.5)	0.628
Hematologic ward	19	(9.8)	8	(11.1)	11	(9.1)	0.685
ICU ward	107	(55.4)	30	(41.7)	77	(63.6)	0.004
Comorbidities							
Cardiovascular disease	77	(39.9)	23	(31.9)	54	(44.6)	0.095
Chronic pulmonary disease	14	(7.3)	5	(6.9)	9	(7.4)	1.000
Chronic liver disease	17	(8.8)	4	(5.6)	13	(10.7)	0.296
Chronic renal disease	29	(15.0)	10	(13.9)	19	(15.7)	0.836
Diabetes	66	(34.2)	27	(37.5)	39	(32.2)	0.531
Malignancy	95	(49.2)	34	(47.2)	61	(50.4)	0.766
CCI score, mean ± SD	4.2 ± 3.1		3.9 ± 3.1		4.4 ± 3.1		0.246
Risk factors							
Neutropenia	16	(8.3)	1	(1.4)	15	(12.4)	0.006
Parenteral nutrition use	55	(28.5)	19	(26.4)	36	(29.8)	0.742
CVC placement	181	(93.8)	67	(93.1)	114	(94.2)	0.765
Urinary catheter	128	(66.3)	39	(54.2)	89	(73.6)	0.007
Steroid therapy	36	(18.7)	14	(19.4)	22	(18.2)	0.850
Chemotherapy	51	(26.4)	16	(22.2)	35	(28.9)	0.399
Prior antibiotic use	167	(86.5)	61	(84.7)	106	(87.6)	0.664
Prior azole exposure	18	(9.3)	9	(12.5)	9	(7.4)	0.307
Clinical severity							
Septic shock	86	(44.6)	13	(18.1)	73	(60.3)	<0.001
Thrombocytopenia	61	(31.6)	7	(9.7)	54	(44.6)	<0.001
SOFA score, mean ± SD	8.5 ± 5.3		4.8 ± 4.0		10.7 ± 4.8		<0.001
Pitt bacteremia score, mean ± SD	3.2 ± 3.2		1.7 ± 1.9		4.1 ± 3.4		<0.001
Source of candidemia							
Primary/unknown	16	(8.3)	4	(5.6)	12	(9.9)	0.419
Intravascular catheter-related	103	(53.4)	45	(62.5)	58	(47.9)	0.054
Abdominal	54	(28.0)	15	(20.8)	39	(32.2)	0.099
Urinary tract	14	(7.3)	5	(6.9)	9	(7.4)	1.000
Others^*a*^	6	(3.1)	3	(4.2)	3	(2.5)	0.673
*Candida* species (*n* = 195)^*b*^							
*Candida albicans*	75	(38.9)	30	(41.7)	45	(37.2)	0.545
*Candida glabrata*	42	(21.8)	12	(16.7)	30	(24.8)	0.210
*Candida tropicalis*	52	(26.9)	13	(18.1)	39	(32.2)	0.043
*Candida parapsilosis* complex^*c*^	17	(8.8)	11	(15.3)	6	(5.0)	0.019
Others^*d*^	9	(4.7)	6	(8.3)	3	(2.5)	0.081
Number of bacterial isolated							0.827
Infected with one bacterial isolate	160	(82.9)	61	(84.7)	99	(81.8)	–^*g*^
Infected with two bacterial isolates	28	(14.5)	9	(12.5)	19	(15.7)	–
Infected with three bacterial isolates	5	(2.6)	2	(2.8)	3	(2.5)	–
MDROs	35	(18.1)	7	(9.7)	28	(23.1)	0.021
Management							
CVC removal	109	(56.5)	56	(77.8)	53	(43.8)	<0.001
Source control	43	(22.3)	21	(29.2)	22	(18.2)	0.107
Empirical therapy							
Empirical antifungal therapy^*e*^	89	(46.1)	37	(51.4)	52	(43.0)	0.297
Triazole-based	52/89	(58.4)	26/37	(70.2)	26/52	(50.0)	–
Echinocandin-based	36/89	(40.4)	11/37	(29.7)	25/52	(48.1)	–
Empirical antibiotics therapy	163	(84.5)	61	(84.7)	102	(84.3)	1.000
Empirical appropriate antibiotics	54/163	(33.1)	26/61	(42.6)	28/102	(27.4)	0.048
EQUAL score, mean ± SD	8.7 ± 3.9		10.7 ± 3.9		7.5 ± 3.3		<0.001

^*a*^ Other sources: endocarditis (2), osteomyelitis (1) or two sources (3), including abdominal/urinary tract (1), intravascular catheter-related/abdominal (2).

^*b*^ Two patients with mixed *Candida* infections:*C. albicans/C. glabrata* (1) and *C. glabrata/C. tropicalis *(1).

^*c*^ *C. parapsilosis* complex: *C. parapsilosis *(14), *C. orthopsilosis* (2), and *C. metapsilosis* (1).

^*d*^ Others: *C. guilliermondii* (2),* C. lusitaniae* (2),* C. duobushaemulonii*(2),* C. pelliculosa* (2)* and C. ciferrii* (1)*.*

^*e*^ Include empirical amphotericin B-based regimen (1).

^*f*^ ICU, intensive care unit; CCI, Charlson Comorbidity Index; SOFA, Sequential Organ Failure Assessment; CVC, central venous catheter; MDROs, multidrug-resistant organisms; EQUAL, European Confederation of Medical Mycology Quality of Clinical Candidaemia Management.

^*g*^ “–”, not applicable.

**TABLE 2:** Univariable and multivariable analyzes of factors associated with 30-day mortality in patients with mixed *Candida*/bacterial bloodstream infections^*a*^^,^^*b*^

	Univariable analysis			Multivariable analysis		
Variables (*P* < 0.05)	OR	95% CI	*P*-value	OR	95% CI	*P*-value
ICU ward	2.45	1.35–4.45	0.003	–^*c*^	–	–
Neutropenia	10.05	1.30–77.77	0.027	5.28	0.45-62.09	0.186
Urinary catheter	2.34	1.27–4.35	0.006	–	–	–
Septic shock	6.90	3.42–13.93	<0.001	–	–	–
Thrombocytopenia	7.48	3.17–17.65	<0.001	3.12	0.86–11.23	0.084
SOFA score	1.33	1.22–1.44	<0.001	1.27	1.11–1.45	0.001
Pitt bacteremia score	1.38	1.21–1.57	<0.001	1.32	1.03–1.68	0.028
*C. tropicalis*	2.16	1.06–4.40	0.034	2.47	0.89–6.82	0.081
*C. parapsilosis* complex	0.29	0.10–0.82	0.020	–	–	–
MDROs	2.80	1.15–6.79	0.023	–	–	–
CVC removal	0.22	0.12–0.43	<0.001	0.38	0.15–1.17	0.091
Empirical appropriate antibiotics	0.52	0.27–0.99	0.046	0.31	0.10–0.95	0.040
EQUAL score	0.79	0.72–0.86	<0.001	0.72	0.62–0.85	<0.001

^*a*^ Goodness-of-fit *P* = 0.732.

^*b*^ OR, odds ratio; CI, confidence interval; ICU, intensive care unit; SOFA, Sequential Organ Failure Assessment; CVC, central venous catheter; MDROs, multidrug-resistant organisms; EQUAL, European Confederation of Medical Mycology Quality of Clinical Candidaemia Management.

^*c*^ “–”, not applicable.

## DISCUSSION

The current study assessed the impact of mixed candidemia and bacteremia on clinical outcomes, focusing on the combinations of *Candida* and bacterial species linked to mortality and identifying mortality predicting factors. We found that mixed *Candida*/bacterial BSIs among 25.2% of candidemia cases resulted in more severe clinical conditions and higher mortality than mono-candidemia, particularly if involving MDROs and *C. tropicalis*. The key predictors of mortality include disease severity, appropriate use of empirical antibiotics, and appropriate management of candidemia.

The incidence of mixed *Candida*/bacterial BSIs in this study accounted for 25.2% of all candidemia cases. This incidence falls within the previously reported range of 18%–56%, representing a significant proportion of candidemia cases (5–10). The variation in incidence rates can likely be attributed to differences in study inclusion criteria. There is no consensus on the time interval between the detection of *Candida* and bacteria in blood cultures of mixed *Candida*/bacterial BSIs. Most studies agree that the detection of >1 pathogen within 24 h to 1 week should be considered a polymicrobial event, and previous studies have commonly used a 48 h to 1-week criterion (5–9, 15, 20, 21).

In the presented study, we found a significantly higher 30-day mortality rate of 62.7% in patients with mixed *Candida*/bacterial BSIs than in those with mono-candidemia. This finding is consistent with a recent study that reported mortality rates for mixed infections significantly exceeded those for mono-candidemia at 28 days and 60 days (9). Interestingly, we noted that shorter intervals between the detection of *Candida* and bacteria in blood cultures corresponded to higher mortality rates in patients with mixed BSIs (Fig. 1B). Mortality rates peaked as both pathogens were isolated from the same blood culture set within the same 24 h period, suggesting a particularly aggressive or severe infection, possibly from the same source. This may reflect a robust invasion by pathogens, likely due to a compromised immune system or rapid pathogen proliferation, leading to severe health consequences (22, 23). While some studies have not identified significant differences in overall mortality and treatment failure rates, severe complications such as septic shock, multiple organ failure, and prolonged stay in intensive care units remain associated with high mortality rates in cases of mixed *Candida*/bacterial BSIs (5, 6). Moreover, mixed BSIs have been observed to potentially impede the early clearance of candidemia and increase the risk of subsequent bacteremia (10).

The distribution of *Candida* spp. in candidemia varies by region (2). Our analysis corroborates previous research indicating no significant differences in the distribution of major *Candida* species between patients with mixed BSIs and those with candidemia alone (6). However, we identified significant differences in the prevalence of less common *Candida* species, such as *C. guilliermondii* and *C. lusitaniae*, among patients with mixed BSIs compared with those with mono-candidemia. The emergence of these rare yeasts is consistent with the species associated with rare *Candida* infections in Taiwan (24). These findings suggest that regional epidemiology, patient-specific conditions, and unique interactions between *Candida* species and co-infecting bacteria may influence their occurrence.

The bacterial species isolated from patients with mixed *Candida*/bacterial BSIs vary, with previous studies indicating a prevalence of Gram-positive and Gram-negative bacteria ranging from 38.8%–68% and 23.7%–61.2%, respectively. The most common Gram-positive bacteria include CoNS, *Enterococcus* spp., and *Staphylococcus aureus*, while the most frequent Gram-negative bacteria are *K. pneumoniae* and *A. baumannii* (5–7, 9). This study found similar levels of Gram-positive and Gram-negative bacteria, with Gram-positive bacteria being slightly more common (52.8%). This differs from previous studies that typically found the dominance of one type (9, 10). The difference from previous studies may be due to the focus on different patient populations, such as those with cerebrovascular accidents and hematological diseases, leading to an uneven bacterial distribution (9, 10). However, the dominant species, such as CoNS, *Enterococcus* spp., and *Klebsiella* spp., remained consistent.

Mortality rates for BSIs involving MDROs are known to be two to four times higher than those for non-MDRO infections (25). Here, MDROs, especially VRE combined with *Candida* spp., particularly *C. tropicalis*, were associated with high mortality rates (Fig. 3 and S2). Previous research has shown that synergistic and antagonistic trans-kingdom interactions modulate the virulence and pathogenicity of *Candida* and their bacteria (26). However, most of these studies have focused on *C. albicans*, and it remains unclear whether the increased mortality observed in infections involving *C. glabrata* or *C. tropicalis* is similarly influenced by the presence of MDROs. The distinctive pathogenic features of *C. tropicalis*, such as its ability to utilize bacterial GlcNAc for filamentation and its expression of multiple ALS adhesins (27), may contribute to the more severe clinical outcomes seen in mixed *Candida*/bacterial BSIs. Nevertheless, further investigation is needed to clarify the species-specific interactions and pathogenic mechanisms in mixed infections involving different *Candida* and bacterial species.

Management of mixed *Candida*/bacterial BSIs requires a multifaceted approach owing to their complexity and severity. Our findings indicate that appropriate use of empirical antibiotics and effective candidemia management are crucial for reducing mortality. In our study, bacterial growth occurred concurrently with, or prior to, *Candida* species in 74.1% (143/193) of cases, suggesting that empirical antibiotics may be warranted in patients with severe illness or clinical suspicion of polymicrobial infection alongside antifungals. Despite 84.5% of the patients with mixed BSIs receiving empirical antibiotics, only one-third of the prescriptions were deemed appropriate. This ineffectiveness of empirical treatment can be attributed to the presence of MDROs, especially VRE, and less common bacterial species, such as *Bacteroides* spp. and *S. maltophilia*, which are linked to high mortality rates (Table S2). The choice of appropriate empirical therapy is crucial and should be tailored based on regional epidemiology. Furthermore, we observed that the EQUAL score was vital in patients with mono-candidemia and those with mixed infections (Fig. S3) (17, 18). Lower scores in several domains, particularly those related to antifungal drug therapy and post-diagnosis assessments, correlated with the early and high mortality observed in patients with mixed BSIs (Fig. S4). The development of targeted antibiotic stewardship programs that address the challenges posed by MDROs and other rare pathogens could markedly enhance the appropriateness of empirical antibiotic use (28). Moreover, exploring the synergistic effects of combined antifungal and antibacterial therapies may allow new avenues for improving treatment efficacy against mixed *Candida*/bacterial BSIs.

This study has some limitations that may lead to an underestimation of the actual incidence of blood cultures with synchronous bacteremia and candidemia. First, blood culture, the primary diagnostic method for the detection of bacteremia and candidemia in adults, has a sensitivity range of 60%–90% (29). However, detection rates for systemic *Candida* infections in blood cultures vary significantly from 21% to 71%, largely because of *Candida’s* unique growth properties and typically lower blood concentrations, which may lead to its less frequent detection compared to other bacteria (30). Additionally, the rapid growth of bacteria within these cultures can suppress fungal growth, further complicating *Candida* (31). Advances in molecular microbiology, such as PCR, may enhance diagnostic sensitivity in the future. Second, follow-up blood cultures in our patients with candidemia were not conducted universally (56.7%, 434/766), and some patients died within 7 days of the first positive blood culture, potentially introducing a bias in the reported duration of candidemia in this study. However, the proportions of patients who died within 7 days of the first positive blood culture was not significantly different between the *Candida*/bacterial BSIs and mono-candidemia groups (42.5% [82/193] vs 35.4% [203/573], *P* = 0.085). Third, the retrospective nature of the study, the 5-year gap since data collection ended, advances in diagnostics (e.g., rapid molecular assays), the emergence of new fungal pathogens, such as *C. auris* and other resistant strains (Table S3), and improvements in therapies that enhance host survival may limit the generalizability of our findings.

Overall, mixed *Candida*/bacterial BSIs were associated with a more severe clinical course and significantly higher mortality rates than mono-candidemia. Early empirical treatment, guided by the prevalence of bacterial species and appropriate management of candidemia, may be a key therapeutic strategy. This study highlights the critical need for heightened vigilance, prompt diagnostic efforts, and tailored therapeutic strategies to address the unique challenges associated with mixed *Candida*/bacterial BSIs.

## SUPPLEMENTAL MATERIAL

Supplemental material spectrum.01684-25-s0001.docx Tables S1 to S3; Figures S1 to S4.

ASM does not own the copyrights to Supplemental Material that may be linked to, or accessed through, an article. The authors have granted ASM a non-exclusive, world-wide license to publish the Supplemental Material files. Please contact the corresponding author directly for reuse.
