# Supplementary material for: Clinical insights into mixed Candida and bacterial bloodstream infections: a retrospective cohort study
Source: Microbiol Spectr. 2025 Sep 4;13(10):e01684-25. doi: 10.1128/spectrum.01684-25 (PMC12502710; doi:10.1128/spectrum.01684-25)
Supplement: Supplemental material — Tables S1 to S3; Figures S1 to S4. [file spectrum.01684-25-s0001.docx]

**Supplementary data**

Table S1. Clinical characteristics of 766 patients with candidemia.

| **Characteristic** | **Total**  N=766, n/N (%) | | **Mixed *Candida*/ bacterial BSIs** N=193, n/N (%) | | **Mono-candidemia**  N=573, n/N (%) | | ***p*-value** |
| --- | --- | --- | --- | --- | --- | --- | --- |
| Demographics | | | | | | | |
| Age (year), mean ± SD | 66.23 ± 14.80 | | 66.25 ± 15.36 | | 66.22 ± 14.62 | | 0.979 |
| Male, sex | 459 | (59.9) | 99 | (51.3) | 360 | (62.8) | 0.005 |
| Surgical ward | 149 | (19.5) | 27 | (14.0) | 122 | (21.3) | 0.027 |
| Medical ward | 175 | (22.8) | 40 | (20.7) | 135 | (23.6) | 0.488 |
| Hematologic ward | 116 | (15.1) | 30 | (15.5) | 86 | (15.0) | 0.908 |
| ICU ward | 366 | (47.8) | 107 | (55.4) | 259 | (45.2) | 0.016 |
| Co-morbidities | | | | | | | |
| Cardiovascular disease | 318 | (41.5) | 77 | (39.9) | 241 | (42.1) | 0.613 |
| Chronic pulmonary disease | 69 | (9.0) | 14 | (7.3) | 55 | (9.6) | 0.384 |
| Chronic liver disease | 60 | (7.8) | 17 | (8.8) | 43 | (7.5) | 0.539 |
| Chronic renal disease | 89 | (11.6) | 29 | (15.0) | 60 | (10.5) | 0.092 |
| Diabetes | 279 | (36.4) | 66 | (34.2) | 213 | (37.2) | 0.490 |
| Malignancy | 409 | (53.4) | 95 | (49.2) | 314 | (54.8) | 0.183 |
| CCI score, mean ± SD | 4.43 ± 3.11 | | 4.22 ± 3.08 | | 4.50 ± 3.12 | | 0.280 |
| Risk factors |  |  |  |  |  |  |  |
| Neutropenia | 56 | (7.3) | 16 | (8.3) | 40 | (7.0) | 0.526 |
| Parenteral nutrition use | 242 | (31.6) | 55 | (28.5) | 187 | (32.6) | 0.325 |
| CVC placement | 668 | (87.2) | 183 | (94.8) | 485 | (84.6) | <0.001 |
| Urinary catheter | 434 | (56.7) | 128 | (66.3) | 306 | (53.4) | 0.002 |
| Steroid therapy | 106 | (13.8) | 36 | (18.7) | 70 | (12.2) | 0.030 |
| Chemotherapy | 186 | (24.3) | 51 | (26.4) | 135 | (23.6) | 0.438 |
| Prior use of antibiotics | 667 | (87.1) | 167 | (86.5) | 500 | (87.3) | 0.804 |
| Prior azole exposure | 63 | (8.2) | 18 | (9.3) | 45 | (7.9) | 0.545 |
| Clinical severity | | | | | | | |
| Septic shock | 272 | (35.5) | 86 | (44.6) | 186 | (32.5) | 0.003 |
| Thrombocytopenia | 179 | (23.4) | 61 | (31.6) | 118 | (20.6) | 0.001 |
| SOFA score, mean ± SD | 7.13 ± 5.31 | | 8.53 ± 5.32 | | 6.65 ± 5.22 | | <0.001 |
| Pitt bacteremia score, mean ± SD | 2.42 ± 2.91 | | 3.16 ± 3.16 | | 2.17 ± 2.78 | | <0.001 |
| Source of candidemia | | | | | | | |
| Primary/unknown | 69 | (9.0) | 16 | (8.3) | 53 | (9.2) | 0.772 |
| Intravascular catheter-related | 394 | (51.4) | 103 | (53.4) | 291 | (50.8) | 0.560 |
| Abdominal | 184 | (24.0) | 54 | (28.0) | 130 | (22.7) | 0.144 |
| Urinary tract | 92 | (12.0) | 14 | (7.3) | 78 | (13.6) | 0.021 |
| Others^†^ | 27 | (3.5) | 6 | (3.1) | 21 | (3.7) | 0.825 |

ICU, Intensive Care Unit; CCI, Charlson comorbidity index; SOFA, sequential organ failure assessment.

^†^ Other sources: endocarditis (9), soft tissue/osteomyelitis (5) or 2 sources (13), including intravascular catheter–related/abdominal (7), intravascular catheter–related/urinary tract (3), and abdominal/urinary tract (3)

Table S2. Distribution of empiric antibiotic selection.

|  | **Empiric antibiotics** | | **Empiric appropriate antibiotic** | |
| --- | --- | --- | --- | --- |
| **Bacterial isolates** | n | (n/N, %) | n | (n/N, %) |
| G (+), N=119 | 99 | (83.2) | 21 | (17.6) |
| G (-), N=95 | 81 | (85.3) | 37 | (38.9) |
| G (+) + (-), N=24 | 20 | (83.3) | 4 | (16.7) |
| MDROs, N=35 | 32 | (91.4) | 3 | (8.6) |
| VRE, N=26 | 24 | (92.3) | 0 | (0.0) |
| CRGNB, N=5 | 5 | (100.0) | 2 | (40.0) |
| MRSA, N=4 | 3 | (75.0) | 1 | (25.0) |
| *Enterococcus spp*., N=52 | 46 | (88.5) | 4 | (7.7) |
| *Staphylococcus aureus*, N=7 | 5 | (71.4) | 2 | (28.6) |
| CoNS , N=55 | 45 | (81.8) | 13 | (23.6) |
| *P. aeruginosa*, N=6 | 5 | (83.3) | 4 | (66.7) |
| *A. baumannii*, N=11 | 10 | (90.9) | 3 | (27.3) |
| *Enterobacterales*, N=62 | 52 | (83.9) | 26 | (41.9) |
| *S. maltophilia*, N=8 | 7 | (87.5) | 2 | (25.0) |
| *Bacteroides spp*., N=4 | 4 | (100.0) | 1 | (25.0) |
|  | **Empiric antibiotics** N=163 | | **Empiric appropriate antibiotic** N=54 | |
| **Antibiotics** | n | (n/N, %) | n | (n/N, %) |
| β-lactam/ β-lactamase inhibitors | 64 | (39.3) | 22 | (40.7) |
| Cephalosporins | 66 | (40.5) | 18 | (33.3) |
| Quinolones | 44 | (27.0) | 9 | (16.7) |
| Carbapenems | 61 | (37.4) | 22 | (40.7) |
| Glycopeptides/ lipopeptide | 31 | (19.0) | 13 | (24.1) |
| Tetracyclines | 26 | (16.0) | 13 | (24.1) |
| Aminoglycosides | 1 | (0.6) | 0 | (0.0) |
| Macrolides | 1 | (0.6) | 0 | (0.0) |
| Clindamycin | 1 | (0.6) | 0 | (0.0) |
| Linezolid | 1 | (0.6) | 0 | (0.0) |
| Colistin | 4 | (2.5) | 2 | (3.7) |
| Rifampin | 4 | (2.5) | 1 | (1.9) |
| Trimethoprim/sulfamethoxazole | 6 | (3.7) | 0 | (0.0) |
| Metronidazole | 4 | (2.5) | 1 | (1.9) |

MDROs, multidrug-resistant organisms; CRGNB, carbapenem-resistant gram-negative bacteria; VRE, vancomycin-resistant *Enterococcus*; MRSA, methicillin-resistant *Staphylococcus aureus*; CoNS, coagulase-negative Staphylococci

Table S3. Antifungal susceptibility results for *Candida* isolates in patients with candidemia. (n=447) ^1,2^

|  |  | Anidulafungin | | | Caspofungin | | | Fluconazole | | | Voriconazole | | |
| --- | --- | --- | --- | --- | --- | --- | --- | --- | --- | --- | --- | --- | --- |
|  | n (%) | S | I | R | S | I | R | S | SDD | R | S | I | R |
| *C. albicans* | 211(47.2) | 211(100.0) | 0(0.0) | 0(0.0) | 209(99.1) | 0(0.0) | 2(0.9) | 202(95.7) | 2(0.9) | 7(3.3) | 202 (95.7) | 3(1.4) | 6(2.8) |
| *C. glabrata* | 84(18.8) | 83(98.8) | 0(0.0) | 1(1.2) | 79(94.0) | 4(4.8) | 1(1.2) | 0(0.0) | 78(92.9) | 6(7.1) | 48^a^(33.3) | - | - |
| *C. tropicalis* | 93(20.8) | 88(94.6) | 1(1.1) | 4(4.3) | 90(96.8) | 2(2.2) | 1(1.1) | 50(53.8) | 13(14.0) | 30(32.3) | 29(31.2) | 41(44.1) | 23(24.7) |
| *C. parapsilosis* complex | 48(10.7) | 39(81.3) | 5(10.4) | 4(8.3) | 44(91.7) | 3(6.3) | 1(2.1) | 46(95.8) | 1(2.1) | 1(2.1) | 44(91.7) | 4(8.3) | 0(0.0) |
| *C. guilliermondii* | 2(0.4) | 2(100.0) | 0(0.0) | 0(0.0) | 2(100.0) | 0(0.0) | 0(0.0) | 2^a^(100.0) | 0(0.0) | 0(0.0) | 2^a^(100.0) | 0(0.0) | 0(0.0) |
| *C. lusitaniae* ^a^ | 4(0.9) | 4(100.0) | 0(0.0) | 0(0.0) | 4(100.0) | 0(0.0) | 0(0.0) | 4(100.0) | 0(0.0) | 0(0.0) | 4(100.0) | 0(0.0) | 0(0.0) |
| *C. intermedia* | 1(0.2) | - | - | - | - | - | - | - | - | - | - | - | - |
| *C. pelliculosa* ^a^ | 2(0.4) | 2(100.0) | 0(0.0) | 0(0.0) | 2(100.0) | 0(0.0) | 0(0.0) | 2(100.0) | 0(0.0) | 0(0.0) | 2(100.0) | 0(0.0) | 0(0.0) |
| *C. haemulonii* ^a^ | 1(0.2) | 1(100.0) | - | - | 1(100.0) | - | - | 0(0.0) | - | - | 0(0.0) | - | - |
| *C. ciferrii* | 1(0.2) | - | - | - | - | - | - | - | - | - | - | - | - |

S, susceptible; SDD, susceptible dose dependent; I, intermediate; R, resistant

^a^ Minimal inhibitory concentration breakpoints adopted from epidemiological cutoff values

^1^ CLSI. *Performance Standards for Antifungal Susceptibility Testing of Yeasts,* 3st ed. CLSI supplement M27M44S. Wayne, PA: Clinical and Laboratory Standards Institute; 2022.

^2^ CLSI. *Epidemiological Cutoff Values for Antifungal Susceptibility Testing,* 4nd ed. CLSI supplement M57S. Wayne, PA: Clinical and Laboratory Standards Institute; 2022.

Figure S1. Flowchart


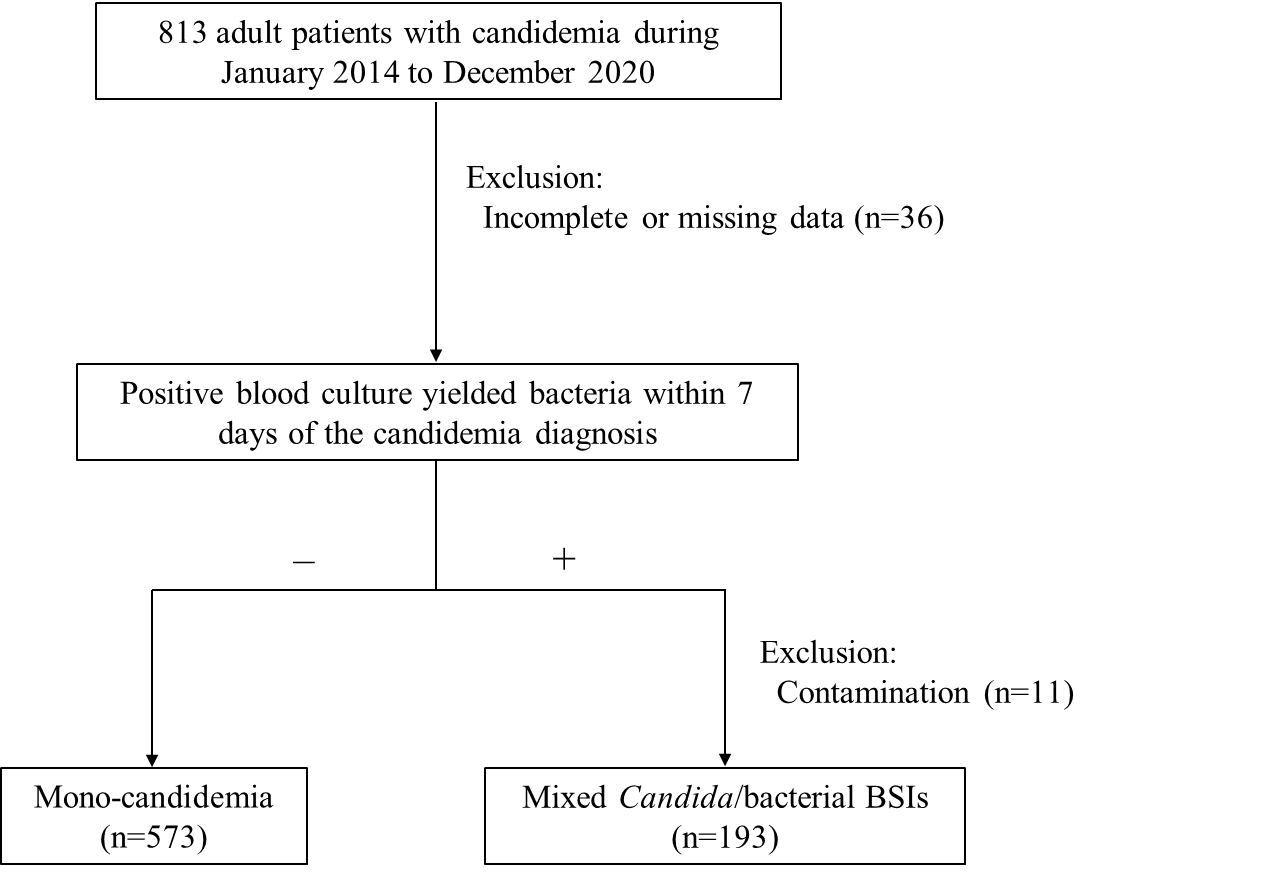


Figure S2. Mortality rates of different Candida species in MDROs-associated candidemia.


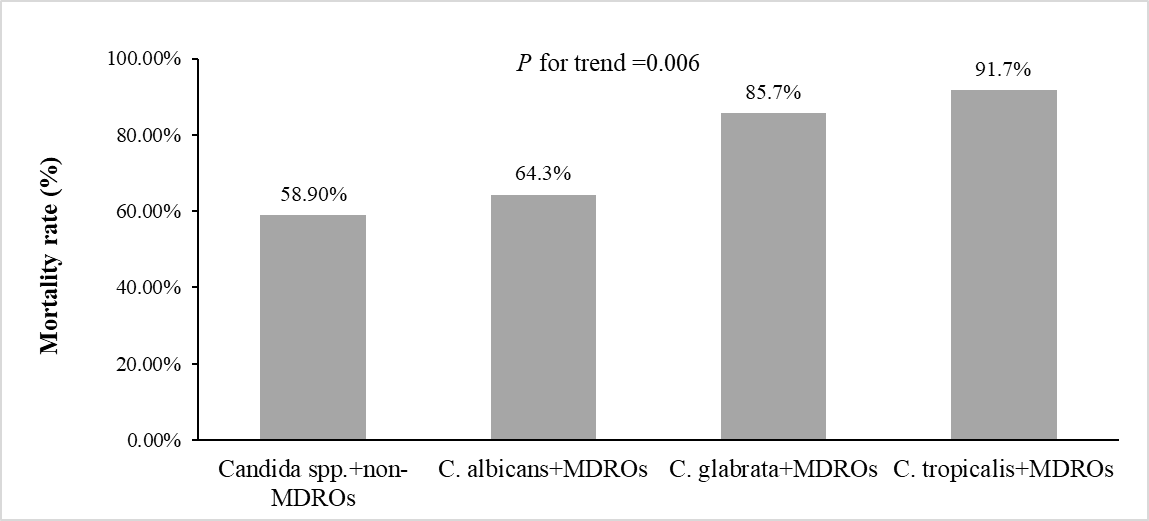


MDROs, multidrug-resistant organisms

Figure S3. Association between EQUAL score and survival rate in patients with mixed *Candida*/bacterial BSIs and mono-candidemia.


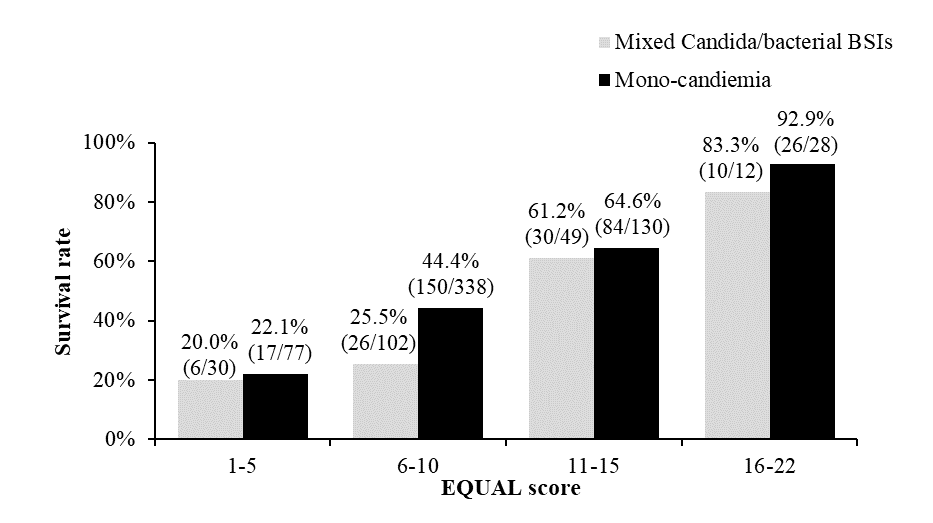


Figure S4. Guideline adherence of EQUAL score in patients with mixed *Candida*/bacterial BSIs.


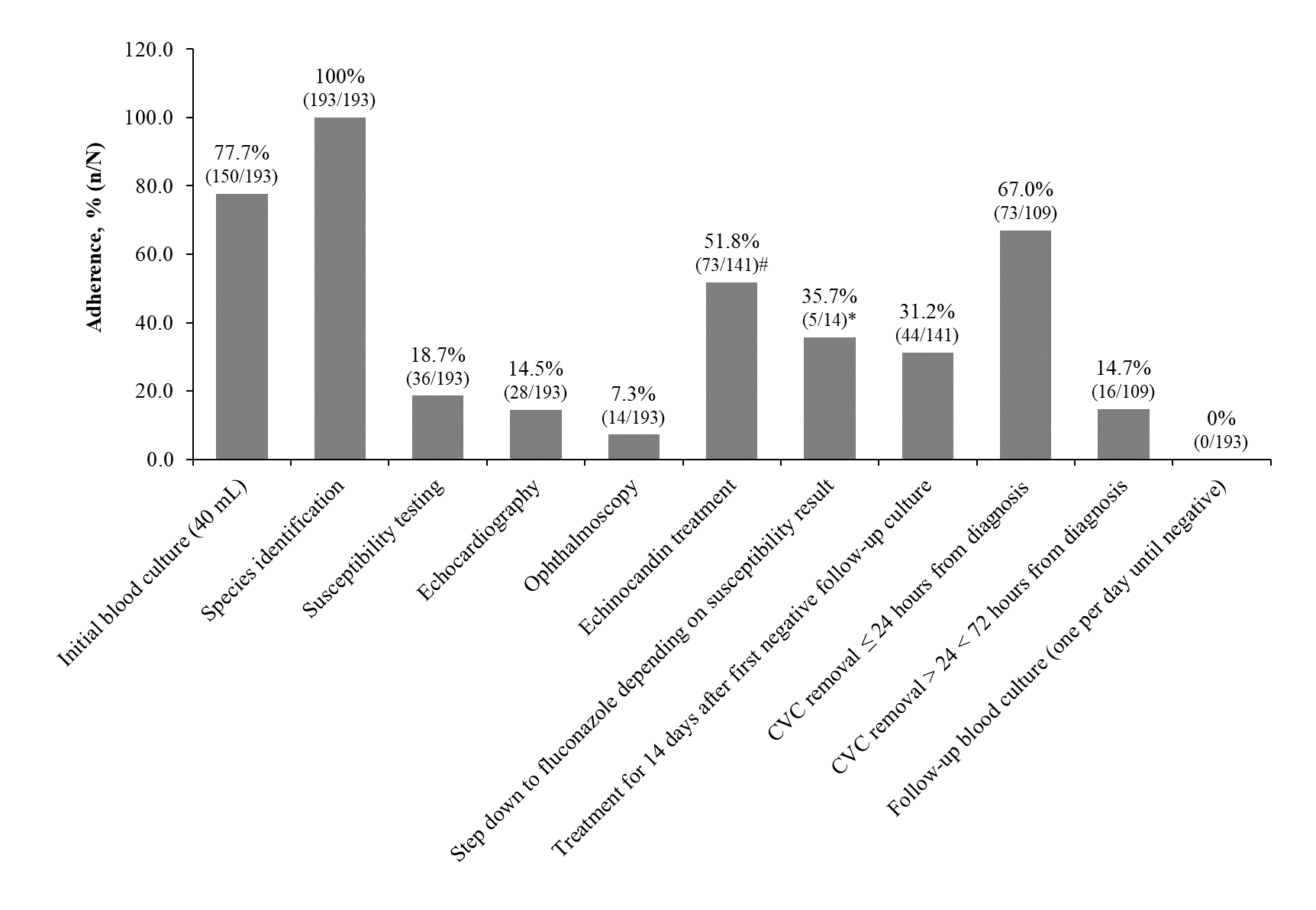


CVC, central vascular catheter

# 141 patients received antifungal therapy.

* 24 antifungal susceptibility testing were conducted on patients receiving echinocandin, and 14 isolates were susceptible to fluconazole.
